# Evidence for a role of spindle matrix formation in cell cycle progression by antibody perturbation

**DOI:** 10.1371/journal.pone.0208022

**Published:** 2018-11-28

**Authors:** Changfu Yao, Chao Wang, Yeran Li, Michael Zavortink, Vincent Archambault, Jack Girton, Kristen M. Johansen, Jørgen Johansen

**Affiliations:** 1 Roy J. Carver Department of Biochemistry, Biophysics, and Molecular Biology, Iowa State University, Ames, Iowa, United States of America; 2 Institut de Recherche en Immunologie et en Cancérologie, Montreal, Canada; Institut de Genetique et Developpement de Rennes, FRANCE

## Abstract

In *Drosophila* it has recently been demonstrated that a spindle matrix in the form of a membrane-less macromolecular assembly embeds the microtubule-based spindle apparatus. In addition, two of its constituents, Megator and Chromator, were shown to function as spatial regulators of spindle checkpoint proteins. However, whether the spindle matrix plays a wider functional role in spatially regulating cell cycle progression factors was unknown. Here using a live imaging approach we provide evidence that a number of key cell cycle proteins such as Cyclin B, Polo, and Ran co-localize with the spindle matrix during mitosis. Furthermore, prevention of spindle matrix formation by injection of a function blocking antibody against the spindle matrix protein Chromator results in cell cycle arrest prior to nuclear envelope breakdown. In such embryos the spatial dynamics of Polo and Cyclin B enrichment at the nuclear rim and kinetochores is abrogated and Polo is not imported into the nucleus. This is in contrast to colchicine-arrested embryos where the wild-type dynamics of these proteins are maintained. Taken together these results suggest that spindle matrix formation may be a general requirement for the localization and proper dynamics of cell cycle factors promoting signaling events leading to cell cycle progression.

## Introduction

The microtubule-based spindle apparatus provides a conserved mechanism to segregate chromosomes during mitosis [1]. However, how this process is coordinated with disassembly and reassembly of nuclear structures during mitotic progression is poorly understood [2]. It is also not clear how enhanced levels of cell cycle regulators and other diffusible molecules are confined within the spindle region in the absence of diffusion barriers following nuclear envelope breakdown (NEB) [3–5]. In *Drosophila* we have identified four nuclear proteins, Skeletor, Chromator, Megator, and EAST from two different nuclear compartments that interact with each other [6–9] and that redistribute during prophase before NEB to form a dynamic, gel-like matrix that embeds the microtubule spindle apparatus, stretching from pole-to-pole [5]. This matrix exists independently of microtubules and the NE and specific interactions between spindle matrix molecules are necessary for complex formation and cohesion [5]. It has been shown that the spindle matrix protein Megator and its human homolog Tpr have an evolutionarily conserved function as spatial regulators of the spindle assembly checkpoint proteins Mad2 and Mps1 [10–12]. However, whether the spindle matrix plays a wider functional role in spatially regulating cell cycle progression factors is unknown. Thus, in order to address how the spindle matrix interacts with cell cycle components we have applied a live imaging approach to determine the relative timing of localization and cross-interactions of these proteins. We provide evidence that a number of key cell cycle proteins such as Cyclin B, Polo, and Ran are co-localized at enriched levels during mitosis after NEB within the spindle matrix and that this localization is independent of microtubules. Furthermore, prevention of spindle matrix formation by injection of a function blocking antibody to the spindle matrix protein Chromator results in cell cycle arrest prior to NEB phenocopying the triple RNAi knockdown of Cyclins A, B, and B3 [13]. Interestingly, in such embryos the dynamic relocalization of Polo and Cyclin B to the nuclear rim and kinetochores is abrogated and Polo is not imported into the nucleus. This is in contrast to colchicine-arrested embryos where the wild-type dynamics of these proteins are maintained. Furthermore, we show that Pdi- and Rnt1-GFP-marked vesicular membranes do not enter the nuclear space defined by the spindle matrix after NEB although they are permeable to microtubules. These studies promise to provide a mechanistic framework for understanding how cell cycle factors are physically confined and organized in the spindle region in organisms with open or semi-open mitosis, allowing for spatial and temporal integration of signaling events leading to mitotic progression and chromosome segregation.

## Materials and methods

### *Drosophila melanogaster* stocks and transgenic flies

Fly stocks were maintained according to standard protocols [14] and Canton S was used for wild-type preparations. Full-length GFP-tagged Chromator constructs under native or *GAL-4* promoter control have been previously characterized [15]. The *H2AvDmRFP1* transgenic line was the gift of Dr. S. Heidmann and has been previously described [16]. The UASp-Gwl-GFP fly line was described in Archambault et al. [17]. The Megator YFP-trap, Rtnl1-GFP-trap, and Pdi-GFP-trap fly lines were obtained from the Kyoto Stock center (stocks 115129, 110624, and 110579, respectively). The Cyclin B-GFP-trap and Polo-GFP-trap fly lines were obtained from the Bloomington Stock Center (stocks 51568 and 51552). For the full-length Megator-mCherry construct under native promoter control, a genomic region from 949 nucleotides upstream of the ATG start codon to the last nucleotide before the stop codon was PCR amplified and fused with an in frame mCherry-tag and inserted into the *pPFHW* vector (DGRC, Vector Barcode: 1125) through gateway recombination [18] using standard techniques [19]. For the Tubulin-mCherry construct, *Tub84B* cDNA (BDGP DGC clone: AT25469) was PCR amplified and fused with an in frame mCherry-tag and inserted into the *pAFHW* vector (DGRC, Vector Barcode: 1119) with an *act5c* promoter through gateway recombination [18] using standard techniques [19]. For the full-length Ran-Venus construct, Ran cDNA (BDGP DGC clone: LD32416) was PCR amplified and inserted into the *pPVW* vector (DGRC, Vector Barcode: 1093) which contains an in frame N-terminal Venus tag through gateway recombination [18] using standard techniques [19]. Transgenic Megator-mCherry, Tubulin-mCherry and Ran-Venus fly lines were generated by P-element transformation by BestGene (Chino Hills, CA). Fly lines expressing combinations of transgenes were generated by standard genetic crosses.

### Timelapse confocal microscopy and injections

Timelapse imaging of the fluorescently-tagged constructs in live syncytial embryos were performed using a Leica TCS SP5 tandem scanning microscope as previously described [5]. In short, 0–1.5 h embryos were collected from apple juice plates, and aged 1 h. The embryos were manually dechorionated, transferred onto a cover slip coated with a thin layer of heptane glue, and covered with a drop of Halocarbon oil 700. Timelapse image sequences of a single z-plane or of z-stacks covering the depth of the mitotic apparatus were obtained using a Plan-apochromat 63X 1.4 NA objective. For colchicine injections, colchicine (Sigma-Aldrich, St. Louis, MO) was dissolved in DMSO to a concentration of 100 mg/ml as a stock solution. The final concentration of colchicine for injection was 1 mg/ml by diluting the stock solution with PEM buffer (80mM Na-PIPES pH 6.9, 1 mM MgCl_2_, 1mM EGTA, 5% Glycerol). Injections of approximately 100–200 pl of 1 mg/ml of colchicine into each embryo were performed with a Narishige Programmable Microinjector IM 300 system connected to the Leica confocal TCS SP5 microscope system as previously described [5,20]. For antibody injections, approximately 100–200 pl of 1μg/μl Chromator mAb 6H11 ascites antibody [7] or GST mAb 8C7 ascites antibody [7] was injected into each embryo. Control injections were performed with DMSO alone or with PEM buffer with 1% DMSO. Fluorescently labeled 70 kDa molecular mass dextrans (Invitrogen, Carlsbad, CA) were injected into syncytial embryos using standard methods as in Yao et al. [5]. All experimental conditions were repeated and observed at least three times.

### Image quantification and analysis

Image processing and quantification were carried out with the ImageJ 1.45 software (NIH, Bethesda, MD) or with Photoshop (Adobe, San Jose, CA). Quicktime movies were generated with Apple Quicktime Pro 7.6.6 (Apple, Cupertino, CA). Average pixel intensities of regions of interest as a function of time were determined in ImageJ and rendered using Microsoft Excel (Microsoft, Redmond, CA). Student's two-tailed t-test statistical analysis was performed using Microsoft Excel (Microsoft, Redmond, CA).

## Results

### Cell cycle arrest by Chromator antibody perturbation

**Fig 1:**
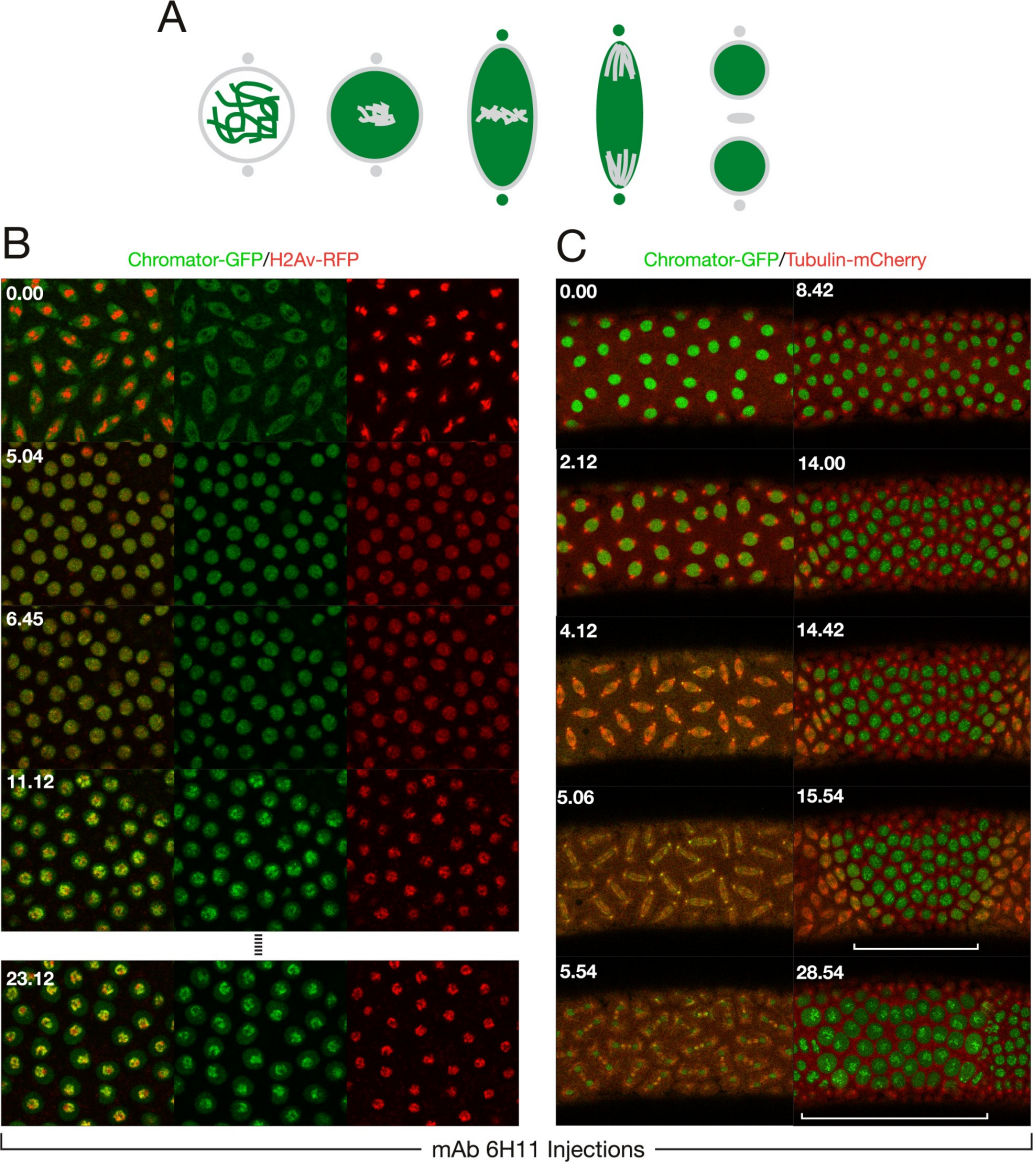
Injection of Chromator mAb 6H11 arrests cell cycle progression prior to NE breakdown. (A) Diagram of the dynamics of Chromator (in green) localization during mitosis in unperturbed embryos based on the results of Yao et al. (Fig 1 in Yao et al. [5]). Chromosomes, the nuclear envelope, centrosomes, and the midbody are in grey and color intensity is proportional to relative protein levels in this and subsequent diagrams. (B) Confocal time-lapse sequence of a mAb 6H11 injected syncytial embryo expressing Chromator-GFP (in green) and H2Av-RFP (in red). The antibody was injected at interphase and the image sequence starts at metaphase of the first cell cycle which progressed normally. However, although the second cell cycle is initiated as indicated by the condensed chromosomes, the normal redistribution of Chromator-GFP away from the chromosomes as well as NEB did not occur (last panel). (C) Confocal time-lapse sequence of a mAb 6H11 injected syncytial embryo expressing Chromator-GFP (in green) and Tubulin-mCherry (in red). This embryo was injected at interphase with a smaller amount of mAb 6H11 where the blocking effect is confined to the immediate vicinity of the injection site (region indicated by the white bracket) due to limited diffusion. The image sequence starts prior to initiation of the first cell cycle. The first mitosis is as in uninjected embryos; however, while the second mitosis is initiated and proceeds normally in the surrounding areas; at the injection site the nuclei arrest prior to NEB with Chromator still present on the condensed chromosomes. Time is indicated in minutes and seconds.

**Fig 2:**
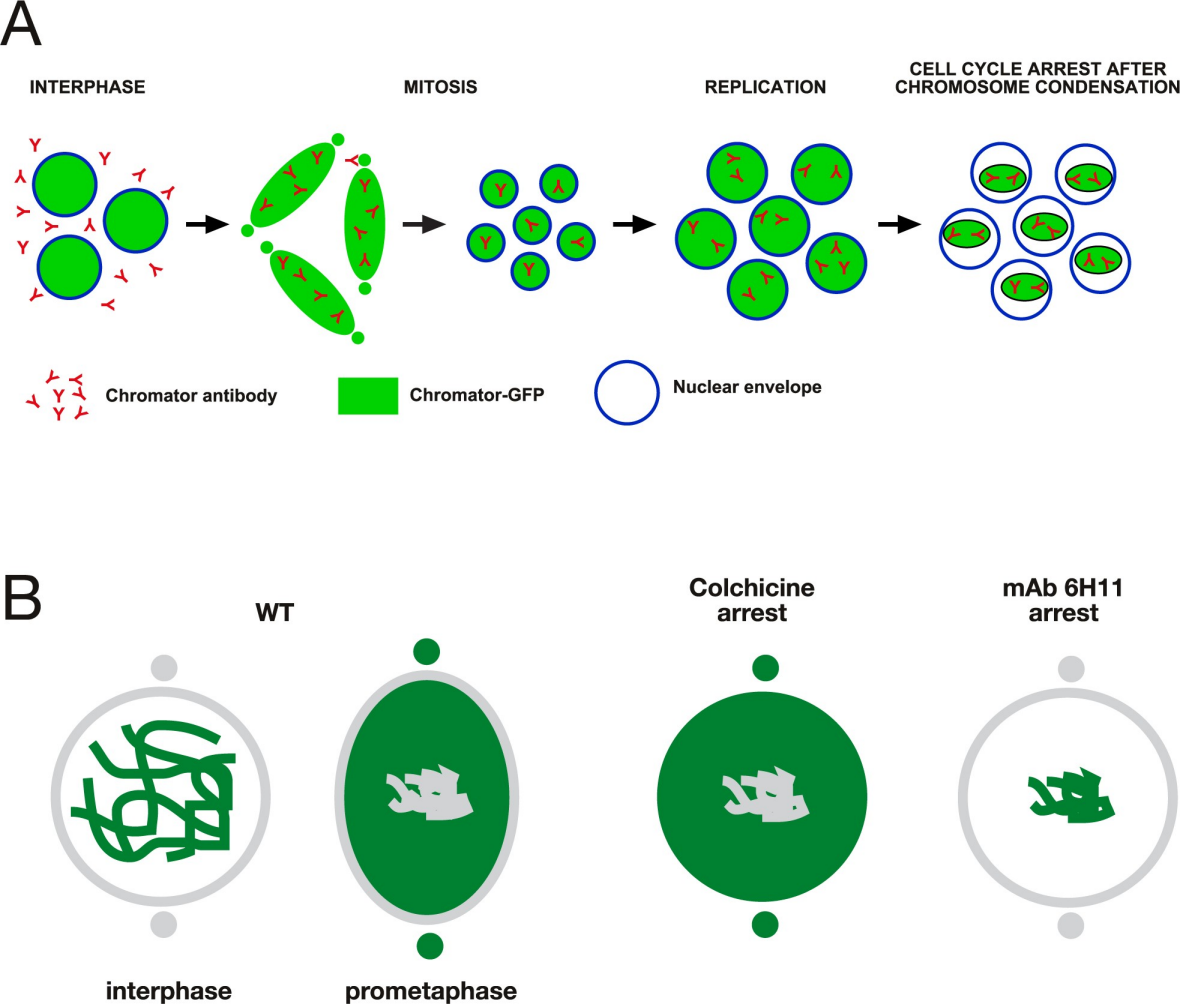
Cell cycle arrest in mAb 6H11 and colchicine arrested embryos. (A) Diagram of proposed model for mAb 6H11-mediated cell cycle arrest. At the time of injection at interphase the antibody does not have access to the Chromator epitope because it is excluded from the chromosomes by the intact nuclear envelope. At the time of NEB of the first cell cycle the antibody is now free to bind to Chromator and this association is maintained as the daughter nuclei become enclosed by the reforming nuclear envelope. That the first cell cycle after NEB proceeds normally even in the presence of the antibody suggests that the presence of mAb 6H11 antibody does not interfere with cell cycle protein function at any point after NEB, with any checkpoint proteins, with cytokinesis, or with formation of the daughter nuclei. However, at the entry of the second cell cycle as indicated by chromosome condensation, the antibody now is bound to Chromator, preventing Chromator dissociation from the chromosomes and spindle matrix formation leading to cell cycle arrest. (B) Comparison of Chromator dynamics in colchicine- and mAb 6H11-arrested embryos. In wild-type, Chromator is localized to the chromosomes at interphase and relocalizes to the spindle matrix and centrosomes at prometaphase and metaphase. In colchicine-arrested embryos Chromator dynamics are as in wild-type embryos (Fig 2 in Yao et al. [5]); however, in mAb 6H11-arrested embryos Chromator remains on the condensed chromosomes, the spindle matrix does not form, and NEB does not occur.

**Fig 3:**
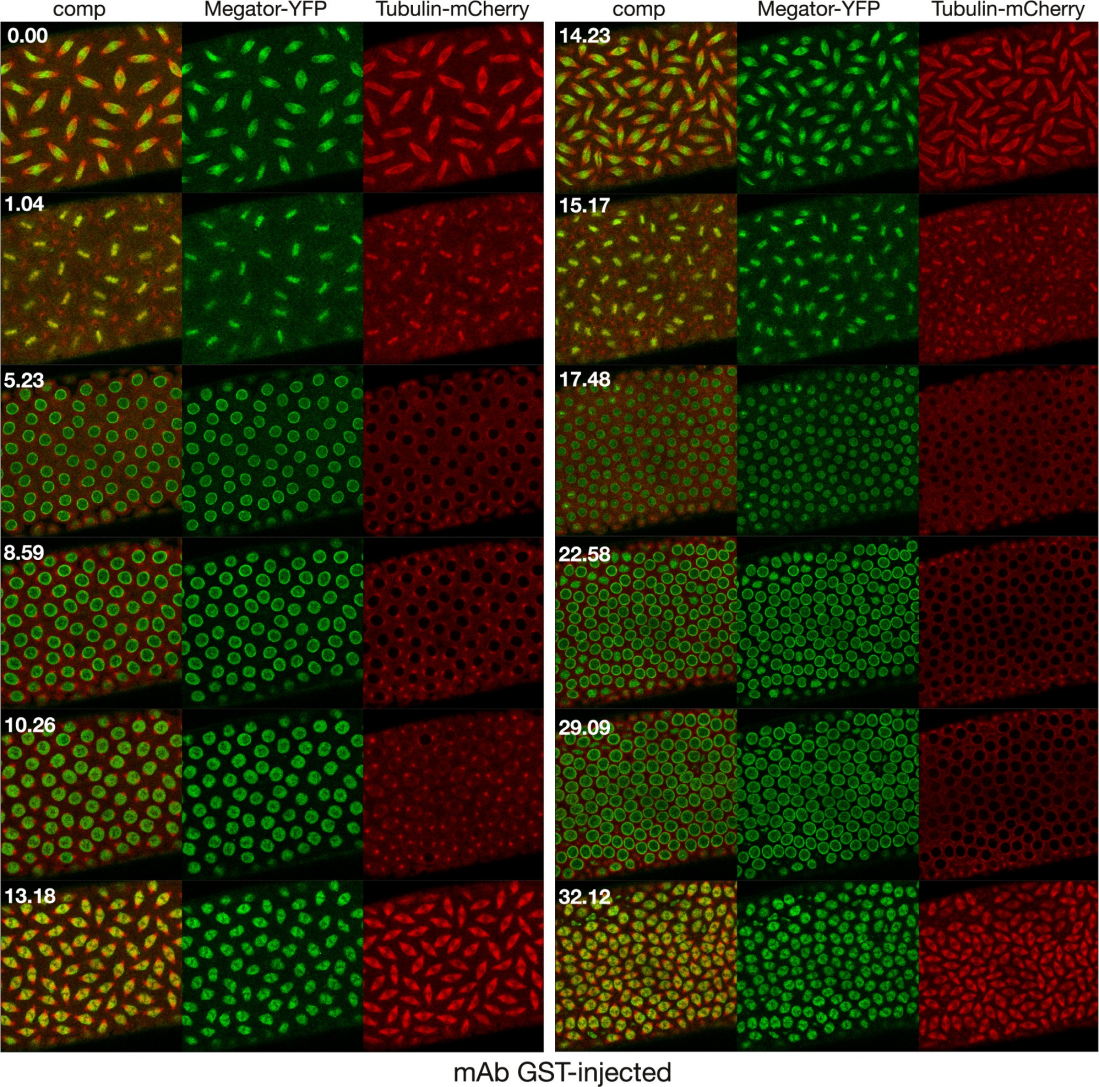
Confocal time-lapse sequence of an α-GST mAb injected control embryo expressing Megator-YFP (in green) and Tubulin-mCherry (in red). The antibody was injected at interphase and the image sequence starts at metaphase of the first cell cycle after the injection. The embryo completed two complete mitotic cycles and initiated a third without any observable defects as compared to wild-type embryos. Time is indicated in minutes and seconds.

One effective way of addressing a protein's function in a given process is to prevent it from performing its role, which can be done through blocking with antibody binding [20,21]. The early *Drosophila* embryo is a particularly advantageous system to employ these strategies for the study of nuclear division, since it consists of a syncytium of nuclei that are readily accessible to molecules injected into the embryo [20]. However, it should be noted that during interphase the nuclear envelope will prevent free access into the nucleus of >40 kDa proteins [22] such as antibodies until NEB occurs. Thus, in order to identify a function blocking antibody that prevents spindle matrix formation we assayed candidate antibodies to known spindle matrix proteins by injecting them into syncytial embryos expressing fluorescently-tagged proteins at interphase and observing the effects by timelapse imaging. The results showed that injection of Chromator mAb 6H11 into embryos led to cell cycle arrest as illustrated in Fig 1. We have previously shown that Chromator is localized to chromosomes during interphase but reorganizes away from the chromosomes as they begin to condense to partake in spindle matrix formation prior to NEB [5] (Fig 1A). After NEB Chromator embeds the forming microtubule-based spindle apparatus as part of the spindle matrix while also translocating to the centrosomes [5] (Fig 1A). At anaphase and telophase Chromator dynamics closely mirror those of the microtubules before relocalizing back to the chromosomes as the daughter nuclei form [5] (Fig 1A). When Chromator mAb 6H11 is injected into a syncytial embryo expressing Chromator-GFP and histone H2Av-RFP at interphase, the first round of mitosis is unaffected; however, the following cycle arrests prior to NEB (Fig 1B; S1 Movie). Although the chromosomes do condense indicating entry into prophase, the normal redistribution of Chromator from the chromosomes to the spindle matrix does not occur (Fig 1B; S1 Movie). That only the second mitosis is affected is likely due to the antibody being prevented access to and binding of Chromator until after NEB of the first mitosis (Fig 2A). The function-blocking activity of mAb 6H11 appears to be specific to preventing spindle matrix formation: division proceeds normally throughout the first mitosis after NEB and mAb 6H11 antibody binding to Chromator. This suggests that the presence of mAb 6H11 antibody does not interfere with cell cycle protein function at any point after NEB, with any checkpoint proteins, with cytokinesis, or with formation of the daughter nuclei. Fig 1C and S2 Movie shows an example of a syncytial embryo expressing Chromator-GFP and Tubulin-mCherry injected with a smaller amount of mAb 6H11 where the blocking effect is confined to the immediate vicinity of the injection site due to limited diffusion. The first mitosis is as in uninjected embryos; however, while the second mitosis is initiated and proceeds normally in the surrounding areas; at the injection site the nuclei arrest prior to NEB with Chromator still present on the condensed chromosomes (Fig 1C and S2 Movie). Moreover, the results support that replication is unaffected since mAb 6H11-arrested nuclei increased in size after entering S phase (Fig 1B and 1C; S1 and S2 Movies). We quantified this aspect by measuring the diameter in pixels of nuclei just after the first cell cycle was completed in Fig 1C (at the 8 min 42 s time point) and comparing it to the diameter of mAb 6H11-arrested nuclei within the white bracket of the 28 min 54 s time point. The results showed that average nuclear diameter increased 42% from 26.8±0.5 pixels (n = 11) to 38.0±0.9 pixels (n = 12). This increase was statistically significant with a P-value less than 0.0001 (Student's two-tailed t-test). In contrast, the average diameter of nuclei not inhibited by mAb 6H11 outside the white bracket at the 28 min 54 s time point decreased 18% to 22.1±0.5 pixels (n = 11). This decrease was statistically significant with a P-value less than 0.0001 (Student's two-tailed t-test). Thus, taken together these observations indicate that the 6H11 antibody very specifically blocks Chromator relocalization away from chromosomes, preventing spindle matrix formation. This effect is very robust and in 15 experiments with Chromator-GFP and H2Av-RFP or Tubulin-mCherry expressing embryos no further progression was observed from this point in observations of up to 45 min. The normal duration of cell cycles at these syncytial stages is approximately 10 min [20]. Control embryos injected with GST antibody underwent normal mitosis indistinguishable from uninjected preparations for at least 3 consecutive cycles (Fig 3; S3 Movie).

The cell cycle arrest mediated by mAb 6H11 is very different from that observed by colchicine arrest as illustrated in Fig 2B. We have previously shown [5] that after tubulin depolymerization by colchicine, Chromator still relocalizes from the chromosomes to the spindle matrix (Fig 2B). However, in the absence of microtubule spindle formation the Chromator-defined matrix did not undergo any dynamic changes but instead statically embedded the condensed chromosomes for extended periods (>20 min) even though the NEB has taken place. Moreover, we showed that there is no diffusion barrier for dextrans up to 2000 kDa [5]. Thus, as a general paradigm spindle matrix or spindle matrix-dependent proteins will be considered as such in the present paper if they are localized within the matrix in colchicine arrested embryos. This includes unpolymerized tubulin which accumulates co-extensively with the matrix relative to the levels outside the nuclear space [5]. It has been hypothesized that this enrichment is due to Chromator's capacity to bind both free and polymerized tubulin [23].

**Fig 4:**
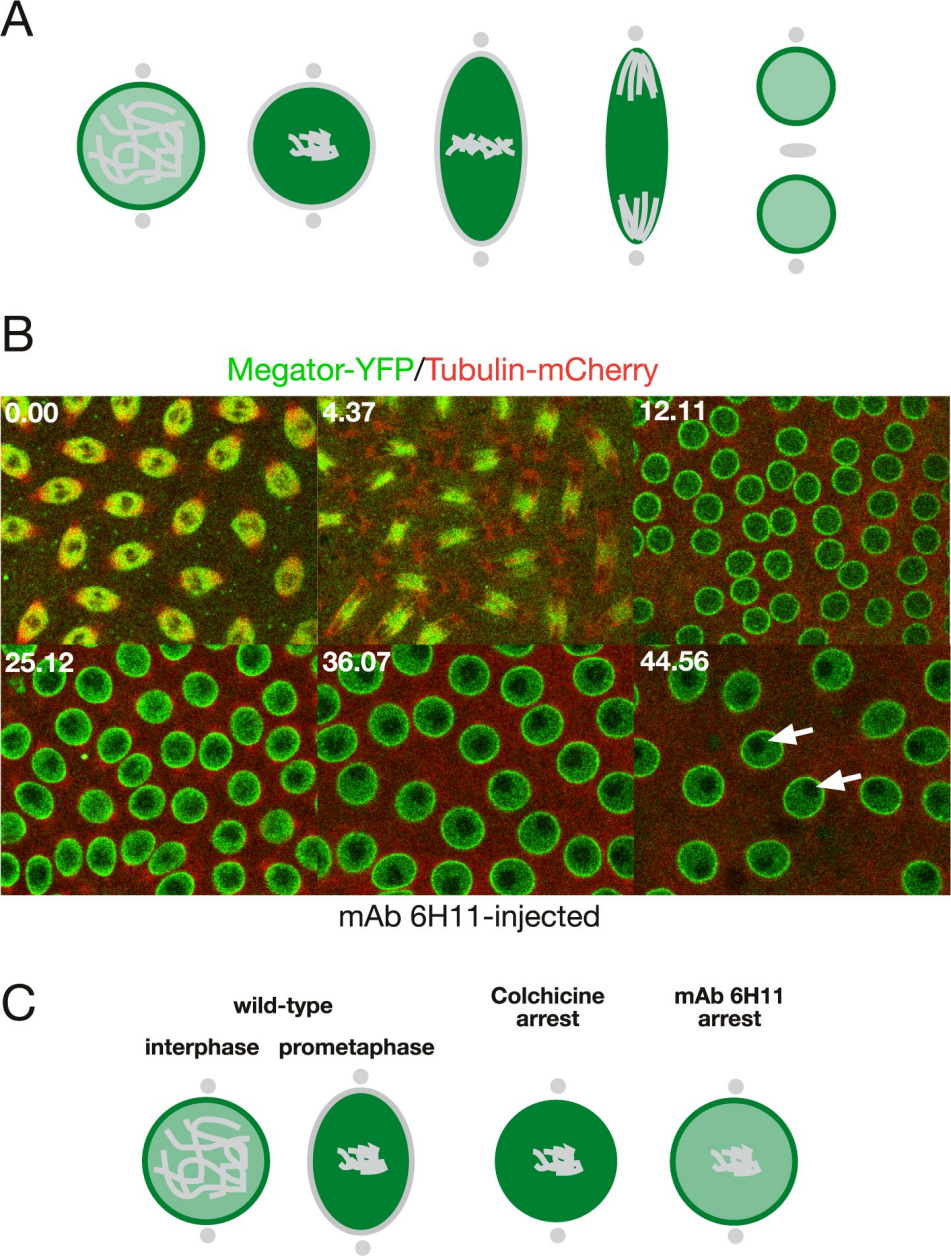
mAb 6H11 injection prevents Megator redistribution from the nuclear envelope during mitosis. (A) Diagram of the dynamics of Megator localization (in green) during mitosis in unperturbed embryos based on the results of Yao et al. (Fig 6 in Yao et al. [5]). (B) Confocal time-lapse sequence of a mAb 6H11 injected syncytial embryo expressing Megator-YFP (in green) and Tubulin-mCherry (in red). The antibody was injected at interphase and the image sequence starts at metaphase of the first cell cycle, which progressed normally, while the second cycle is arrested prior to NEB. The last image was obtained approximately 35 min after the first cell cycle was completed. The chromosomes have condensed (dark regions indicated by white arrows) and Megator-YFP is still present on the NE at a time when it normally would have relocalized to the spindle matrix. Time is indicated in minutes and seconds. (C) Comparison of Megator dynamics in colchicine- and mAb 6H11-arrested embryos. In wild-type, Megator is localized to the nuclear interior and the NE at interphase and relocalizes to the spindle matrix at prophase. In colchicine-arrested embryos Megator dynamics is as in wild-type embryos (Fig 5E in Yao et al. [5]); however, in mAb 6H11-arrested embryos Megator remains at the nuclear rim, the spindle matrix does not form, and NEB does not occur.

To probe whether mAb 6H11-antibody mediated cell cycle arrest may be caused by a general block of spindle matrix formation we injected mAb 6H11 antibody into embryos at interphase expressing Megator-YFP and Tubulin-mCherry (Fig 4). As diagrammed in Fig 4A Megator localizes to the nuclear interior as well as the nuclear rim at interphase and to the spindle matrix at metaphase during mitosis in uninjected embryos [5,8]. However, as illustrated in Fig 4B and S4 Movie, at the point of cell cycle arrest in a mAb 6H11-injected embryo after entry into the second mitosis, as indicated by the condensed chromosomes (dark regions within the nuclei), Megator is still present on the nuclear rim at a time when it would normally have relocalized to the spindle matrix. This is in contrast to colchicine-arrested embryos where Megator translocates to the spindle matrix as in uninjected embryos [5] (Fig 4C). Thus, taken together the findings that mAb 6H11-antibody mediated arrest prevents both Chromator relocalization from the chromosomes as well as Megator relocalization from the NE indicate a general block of spindle matrix formation resulting in cell cycle arrest.

### Cell cycle factors and the spindle matrix

The spindle matrix proteins Chromator and Megator have been demonstrated to function as spatial regulators for spindle checkpoint proteins such as Mad2 and Mps1 [10–12,15]. Furthermore, the effect of Chromator-antibody perturbation of cell cycle progression phenocopies the triple RNAi knockdown of Cyclins A, B, and B3 [13]. This raises the possibility that proper signaling events mediated by these cyclins and other cell cycle proteins are required for initiation of NEB and that this signaling is prevented by perturbation of spindle matrix formation. To address these issues we compared the dynamics of fluorescently-tagged cell cycle factors, e.g. Cyclin B, Ran, and Polo, by timelapse imaging in embryos where mitosis was arrested by either colchicine or mAb 6H11-antibody. Cyclin B, Ran, and Polo were selected for study based on the availability of transgenic fly lines with sufficient fluorescent signal to allow for long term confocal imaging at high frame rates.

#### Cyclin B

**Fig 5:**
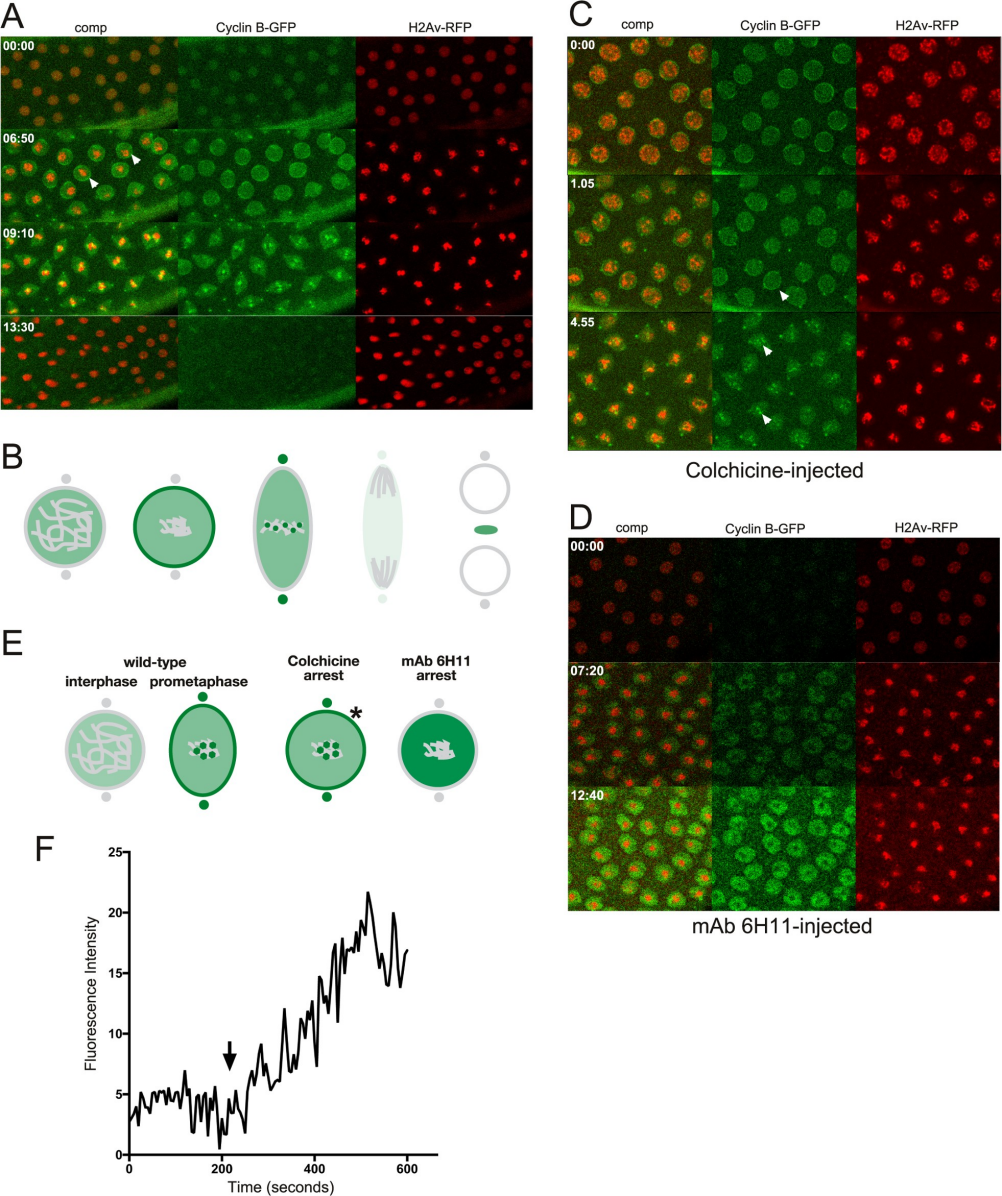
Cyclin B dynamics in un-, colchicine-, and mAb 6H11-injected embryos. (A) Confocal image sequence of the relative dynamics of Cyclin-B-GFP (in green) and H2Av-RFP (in red) during a mitotic cycle in an untreated embryo. Arrowheads point to enhanced Cyclin-B-GFP localization at the nuclear rim at the time of NEB. Time is indicated in minutes and seconds. (B) Diagram of the dynamics of Cyclin B (in green) localization during mitosis in unperturbed embryos. (C) Confocal image sequence of Cyclin-B-GFP (in green) and H2Av-RFP (in red) dynamics during a mitotic cycle in a colchicine-injected embryo. Arrowheads indicate Cyclin B localization to the nuclear rim and kinetochores similar to that observed in untreated embryos. (D) Confocal image sequence of a Cyclin-B-GFP (in green) and H2Av-RFP (in red) expressing embryo injected with Chromator mAb 6H11. The antibody was injected at interphase and the image sequence starts approximately 2 min after the first cell cycle was completed. Although Cyclin-B-GFP levels appear to be continuously increasing within the nucleus there is no indication of its normal enrichment at the nuclear rim, kinetochores of the condensed chromosomes, or centrosomes. (E) Comparison of Cyclin B dynamics in colchicine- and mAb 6H11-arrested embryos. In wild-type Cyclin B is present at low levels in the nuclear interior, accumulates within the spindle matrix at prometaphase and transiently relocates to the nuclear rim, the kinetochores, and the centrosomes. In colchicine-arrested embryos Cyclin B dynamics are as in wild-type embryos; however, in mAb 6H11-arrested embryos Cyclin B-levels increase within the nucleus without localization to the nuclear rim, kinetochores, or centrosomes. The asterisk indicates the transient Cyclin B localization to the nuclear rim just prior to NEB in colchicine-treated embryos. (F) Plot of average pixel density within the nucleus as a function of time for Cyclin B-GFP. Average pixel density for an area outside the nuclei was subtracted at each time point. Increased levels of Cyclin B-GFP accumulate in the nucleus as chromosome condensation commences (arrow).

Cyclin B is a dynamically regulated mitotic cyclin that interacts with Cdk1 and contributes to mitotic progression [24,25]. As illustrated in Fig 5A and S5 Movie and diagrammed in Fig 5B, during the cell cycle in an uninjected embryo, Cyclin-B-GFP accumulates in the nucleus during S phase and is transiently enhanced at the nuclear rim at the time of NEB. During metaphase there is an enrichment at kinetochores and centrosomes before Cyclin B degradation commences during ana- and telophase [26]. In colchicine-injected embryos expressing Cyclin-B-GFP (Fig 5C and S6 Movie) there is enhanced nuclear rim, kinetochore, and centrosome localization, and importantly enrichment in the spindle region is maintained after NEB as verified in dextran injected embryos (S1 Fig and S7 Movie), suggesting interactions with and localization to the spindle matrix. However, in mAb 6H11-cell-cycle-arrested embryos (Fig 5D and S8 Movie), although Cyclin-B-GFP levels appear to be continuously increasing within the nucleus there is no sign of its normal enrichment at the nuclear rim, kinetochores of the condensed chromosomes, or centrosomes. The differences in Cyclin B localization at prometaphase in unperturbed, colchicine-arrested, and mAb 6H11-arrested nuclei are diagrammed in Fig 5E. That Cyclin B-GFP accumulated in mAb 6H11-arrested nuclei as chromosome condensation was initiated is demonstrated in the plot of average pixel density within a nucleus as a function of time (Fig 5F). Taken together these results support the hypothesis that spindle matrix formation is required for Cyclin B localization and dynamics, thus facilitating signaling events leading to NEB and cell cycle progression.

#### Ran

**Fig 6:**
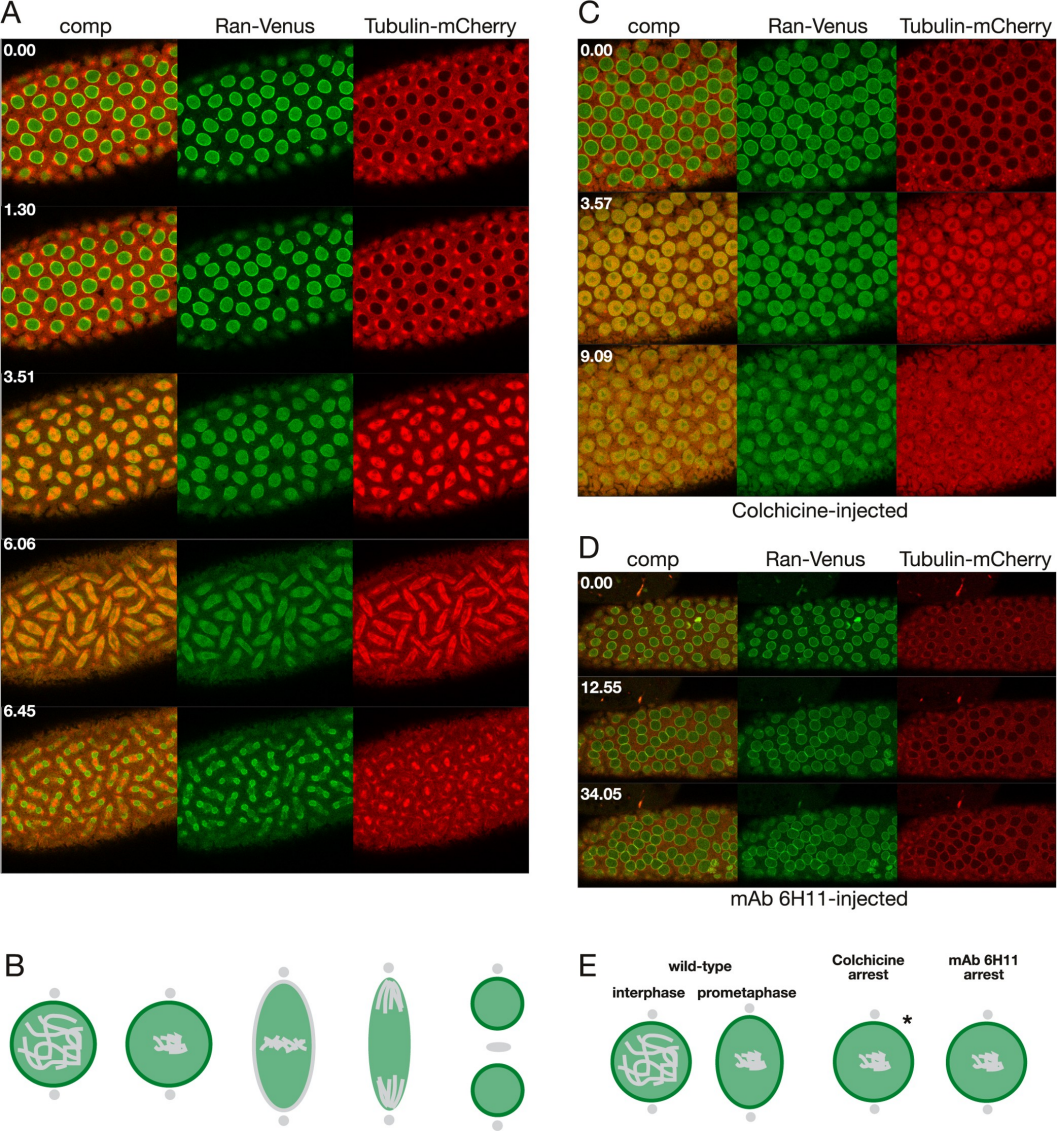
Ran dynamics in un-, colchicine-, and mAb 6H11-injected embryos. (A) Confocal image sequence of the relative dynamics of Ran-Venus (in green) and Tubulin-mCherry (in red) during a mitotic cycle in an untreated embryo. Time is indicated in minutes and seconds. (B) Diagram of the dynamics of Ran (in green) localization during mitosis in unperturbed embryos. (C) Confocal image sequence of Ran-Venus (in green) and Tubulin-mCherry (in red) dynamics during a mitotic cycle in a colchicine-injected embryo. Ran-Venus is maintained within the nuclear domain even after NEB. Note also that after NEB free tubulin accumulates within the spindle matrix at much higher levels than in the surrounding syncytial cytoplasm. (D) Confocal image sequence of a Ran-Venus (in green) and Tubulin-mCherry (in red) expressing embryo injected with Chromator mAb 6H11. The antibody was injected at interphase and the image sequence starts approximately 4 min after the first cell cycle was completed. No changes to the localization of Ran-Venus was observed and it maintained its nuclear rim localization, as there is no NEB. (E) Comparison of Ran dynamics in colchicine- and mAb 6H11-arrested embryos. In wild-type Ran is present at the nuclear interior and at the nuclear rim at interphase as well as at prometaphase prior to NEB. In colchicine-arrested embryos Ran dynamics is as in wild-type embryos; however, in mAb 6H11-arrested embryos there are no changes to the localization of Ran and it maintains its nuclear rim localization, as there is no NEB. The asterisk indicates the transient Ran localization to the nuclear rim just prior to NEB in colchicine-treated embryos.

Another protein important for cell cycle progression is the small GTPase Ran which can form RanGTP/GDP gradients that are maintained even through M-phase [27]. As illustrated in Fig 6A and S9 Movie and diagrammed in Fig 6B, during the cell cycle in an uninjected embryo expressing Ran-Venus together with Tubulin-mCherry, Ran-Venus is present in the nucleus during S phase and at the nuclear rim until NEB. During metaphase the levels of Ran-Venus is maintained and it envelopes the microtubule-based spindle apparatus in a manner similar to that described for the spindle matrix proteins Chromator and Megator [5]. That Ran is a likely spindle matrix component was further confirmed in colchicine-injected embryos (Fig 6C and S10 Movie) where the levels of Ran-Venus is maintained within the nuclear domain even after NEB. Note also that after NEB free tubulin accumulates within the spindle matrix at much higher levels than in the surrounding syncytial cytoplasm as previously described in Yao et al. [5]. In mAb 6H11-cell-cycle-arrested embryos (Fig 6D and S11 Movie) there are no changes to the localization of Ran-Venus and it maintains its nuclear rim localization, as there is no NEB. The localization of Ran at prometaphase in unperturbed, colchicine-arrested, and mAb 6H11-arrested nuclei are diagrammed in Fig 6E. These data suggest that Ran is a spindle matrix constituent and provide a mechanism for how Ran-GTP/GDP gradients can be maintained at M-phase after NEB when there is no diffusion barrier.

#### Polo

**Fig 7:**
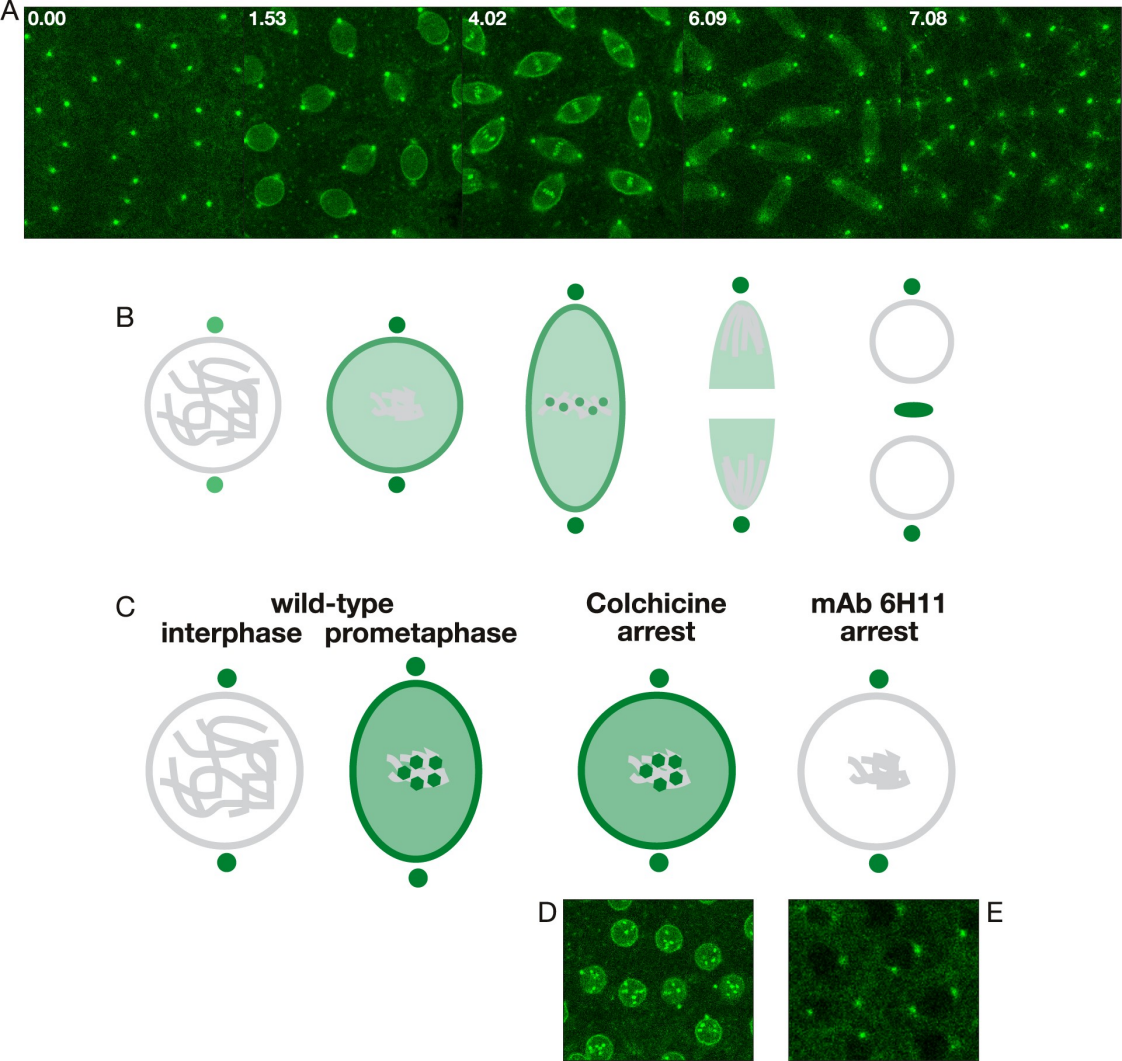
Polo dynamics in un-, colchicine-, and mAb 6H11-injected embryos. (A) Confocal image sequence of Polo-GFP during the cell cycle in an untreated embryo. Time is indicated in minutes and seconds. (B) Diagram of Polo (in green) dynamics during mitosis in unperturbed embryos. (C) Comparison of Polo localization after cell cycle arrest with colchicine- and mAb 6H11-injections, respectively. In wild-type, Polo is present at centrosomes but not in the nuclear interior at interphase; however, it accumulates during pro- and prometaphase and transiently relocalizes to the nuclear rim and the kinetochores. After colchicine-arrest Polo localizes to the spindle matrix, the nuclear rim, and kinetochores as in wild-type preparations. However, in mAb 6H11 arrested embryos Polo does not accumulate in the nucleus and is present only at centrosomes. (D) Confocal image of polo-GFP from an image sequence from a colchicine-arrested embryo showing Polo-GFP enrichment at the nuclear rim and at the kinetochores. (E) Confocal image of Polo-GFP from the end of an image sequence of a mAb 6H11-arrested embryo where Polo-GFP is only present at the centrosomes.

Another crucial kinase for cell cycle progression is Polo [28,29]. As illustrated in Fig 7A and S12 Movie and diagrammed in Fig 7B, during the cell cycle in an uninjected embryo expressing Polo-GFP, Polo-GFP was localized to the centrosomes throughout the cell cycle but was not present or at very low levels within the nucleus at interphase. However, at the onset of mitosis Polo-GFP begins to accumulate in the nucleus with gradual enhanced localization to the nuclear rim just prior to NEB. Furthermore, Polo-GFP shows transient enhanced localization to the kinetochores of the condensed chromosomes aligned at the metaphase plate. Interestingly, in colchicine-cell-cycle-arrested embryos, Polo-GFP undergoes its normal nuclear accumulation as well as its enhanced localization to the nuclear rim and kinetochores (Fig 7C and 7D, S13 Movie). In contrast, in mAb 6H11-arrested embryos where the spindle matrix does not form, Polo does not accumulate in the nucleus and is absent from the nuclear rim and the kinetochores (Fig 7C and 7E, S14 Movie). These findings provide support for the hypothesis that spindle matrix formation is required for proper Polo dynamics and function during the cell cycle as well as for orchestrating the temporal loading of cell cycle factors to various nuclear locations such as the nuclear rim and kinetochores when needed.

#### Greatwall

**Fig 8:**
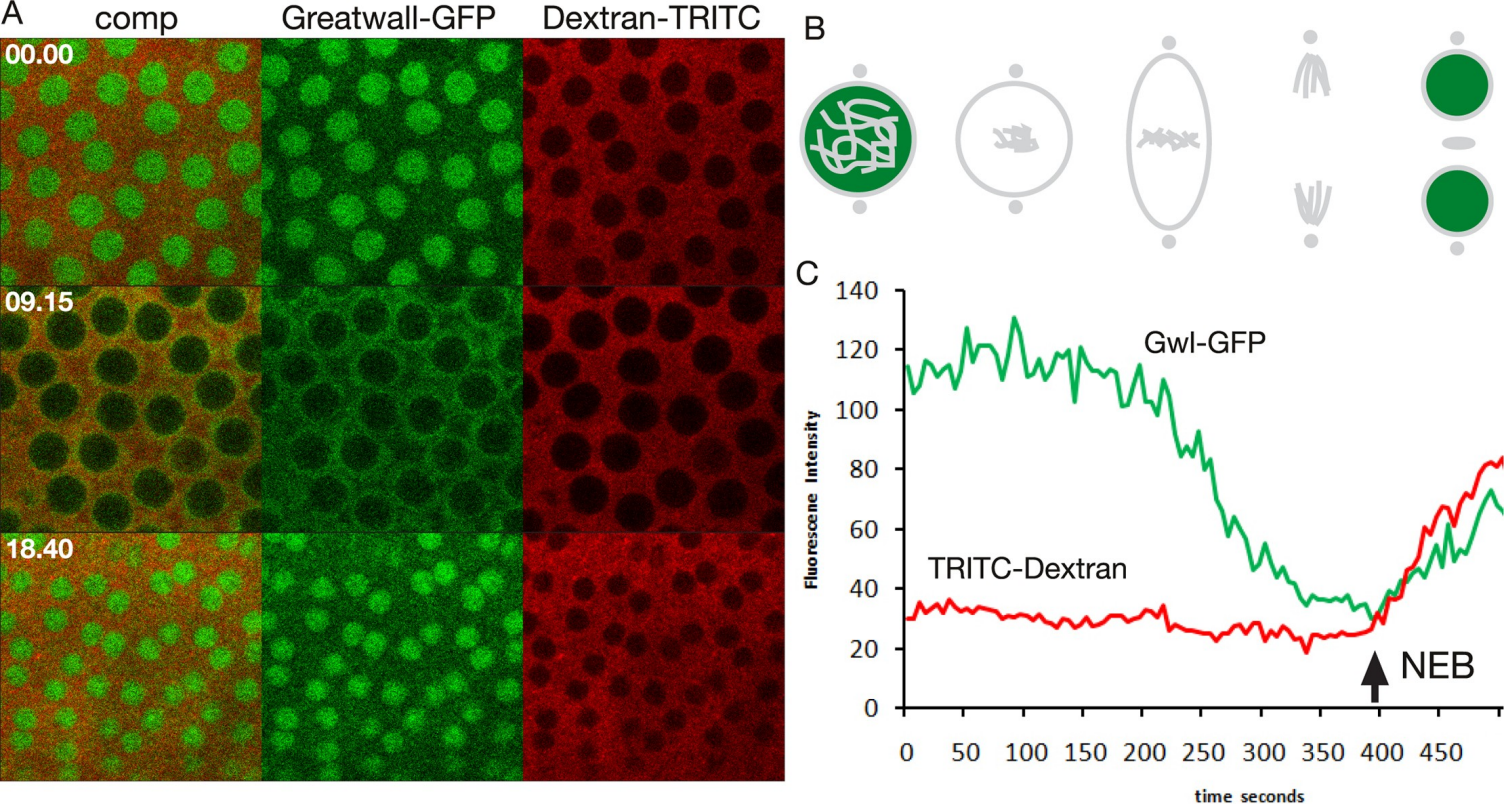
The Greatwall kinase is not a spindle matrix protein. (A) Confocal image sequence of the relative dynamics of Greatwall-GFP and 70 kDa Dextran-TRITC during a mitotic cycle in an unperturbed embryo. Time is indicated in minutes and seconds. (B) Diagram of Greatwall (in green) dynamics during mitosis. (C) Plots of average pixel density within the nucleus as a function of time for Greatwall-GFP and 70 kDa Dextran-TRITC. Greatwall-GFP (Gwl-GFP) is being cleared from the nucleus during prophase prior to NEB (arrow) and coincident with spindle matrix formation.

As an example of a cell cycle regulator that does not localize to the spindle matrix we performed live imaging studies of Greatwall-GFP-expressing embryos injected with 70 kDa Dextran-TRITC (Fig 8A and S15 Movie). The Greatwall kinase is present in the nucleus at interphase [30]; however, prior to spindle matrix formation and NEB it is exported out of the nucleus [30] and does not localize to the spindle matrix during pro-, meta-, or anaphase (Fig 8A and 8B, S15 Movie). That Greatwall-GFP is being cleared from the nucleus prior to NEB and coincident with spindle matrix formation is demonstrated in plots of average pixel density within the nucleus as a function of time for Greatwall-GFP (in green) and 70 kDa Dextran-TRITC (in red) (Fig 8C). The arrow in Fig 8C indicates the timepoint when TRITC-Dextran enters the nucleus indicating NEB. Thus, these experiments support the hypothesis that the spindle matrix is a specific assembly of macromolecules and that some cell cycle factors are incorporated into the matrix whereas others are excluded.

### Endoplasmic reticulum and other membranes are excluded from the spindle matrix even when permeable to microtubules

**Fig 9:**
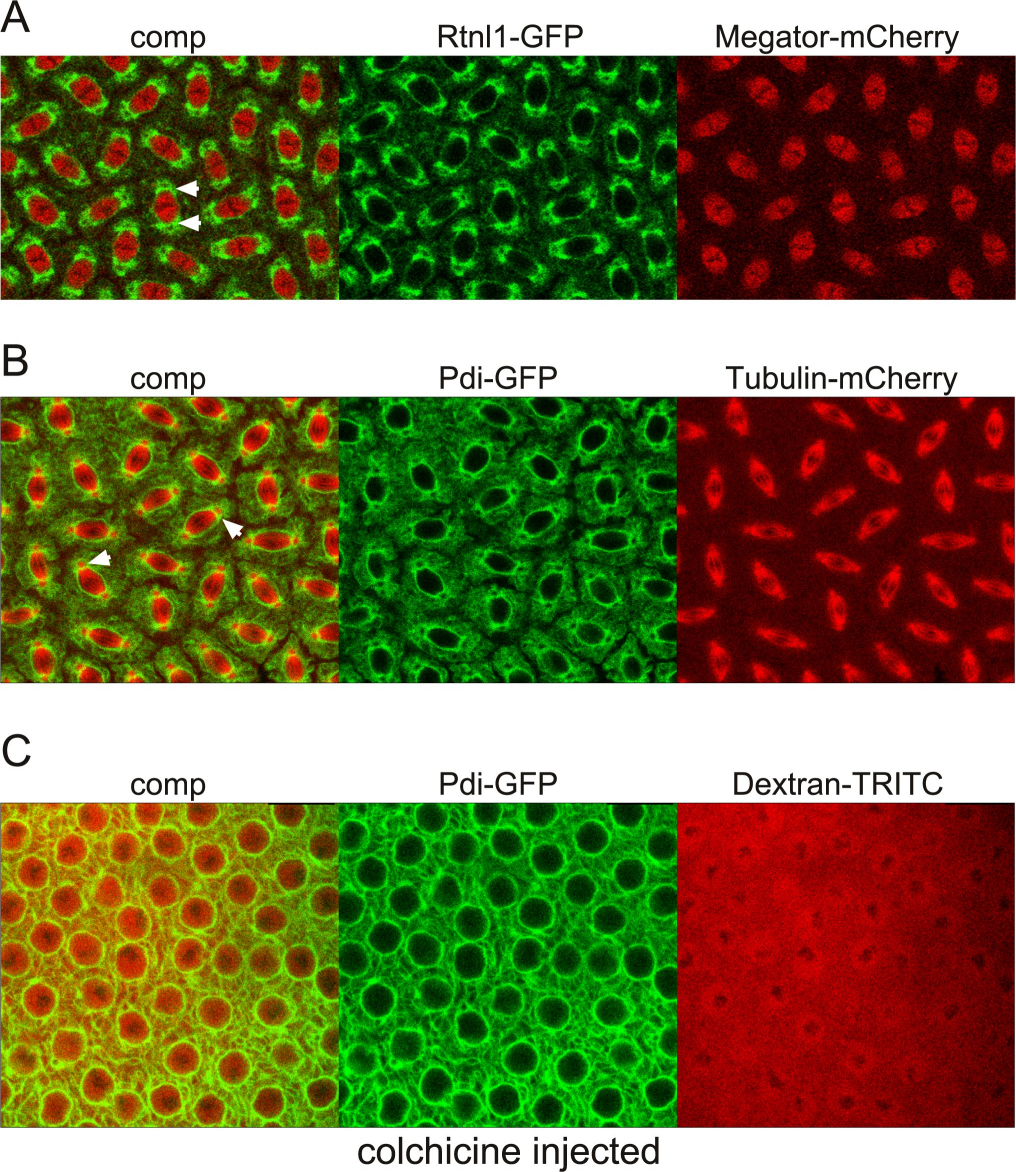
Endoplasmic reticulum and other membranes are excluded from the spindle matrix even when permeable to microtubules. (A) Confocal images at metaphase from a mitotic image sequence from an embryo expressing Rtnl-GFP (in green) and Megator-mCherry (in red). Arrowheads indicate membranes accumulated in the gap between the spindle matrix as represented by Megator-mCherry and the centrosome (black area surrounded by the membranes). (B) Confocal images at metaphase from a mitotic image sequence from an embryo expressing Pdi-GFP (in green) and Tubulin-mCherry (in red). Arrowheads indicate membranes accumulated in the gap between the spindle matrix and the centrosome through which microtubules extend as indicated by the yellow color. (C) Confocal images from a Pdi-GFP expressing embryo injected with 70 kDa Dextran-TRITC as well as with colchicine. After NEB Dextran-TRITC invades the nuclear space; however, Pdi-GFP-labeled membranes are still excluded from the nuclear interior.

During mitosis there is a dramatic reorganization of the endoplasmic reticulum (ER) and other organelles as well as of nuclear membranes [31]. In *Drosophila* syncytial embryos the ER at interphase is spread loosely around the nucleus; however, as mitosis progresses the ER membranes reorganize and accumulate around the nucleus especially around the spindle poles and the centrosomes at metaphase [32]. Interestingly, even as the chromosomes are fully condensed these membranes and other cytoplasmic organelles do not enter the nuclear space where the microtubule based spindle apparatus is forming [31]. Thus, to determine the temporal-spatial relationship of ER membranes with the spindle matrix we performed timelapse imaging of syncytial embryos expressing Rtnl1-GFP [33] and Megator-mCherry (Fig 9A and S16 Movie). Rtnl1 (Reticulon-like 1) is an ER shaping protein embedded in the cytoplasmic face of the ER and it colocalizes with ER structures [32,34]. As illustrated in Fig 9A Rtnl1-GFP labeled membranes have coalesced around the spindle matrix as defined by Megator-mCherry without entering the spindle matrix-occupied space with most of the membranes concentrated in the gap between the centrosomes and the spindle matrix. Interestingly, this accumulation of membranes does not represent a barrier to microtubules emanating from the centrosomes as illustrated in Fig 9B which shows microtubules in relation to membranes at metaphase from a timelapse movie of the dynamic relationship between Tubulin-mCherry and Pdi-GFP in a syncytial embryo (S17 Movie). Pdi (Protein disulfide isomerase) is an ER-lumenal protein [32,35]. That the exclusion of membranes from the nuclear space is independent of microtubules is further illustrated in Fig 9C and S18 Movie which show a Pdi-GFP expressing embryo injected with 70 kDa Dextran-TRITC as well as with colchicine. After NEB Dextran-TRITC invades the nuclear space; however, Pdi-GFP-labeled membranes are still excluded in the absence of polymerized tubulin. Taken together these observations raise the possibility that a function of the spindle matrix may be to help exclude organelles and membranes from the spindle region as well as to assist in positioning them for even distribution around the forming daughter nuclei.

## Discussion

In this study we have determined the relationship of key cell cycle factors e.g. Cyclin B, Ran, and Polo with the spindle matrix using a live imaging approach. During mitosis their spatial localization is highly dynamic with transient loading to various nuclear locations such as the nuclear rim and kinetochores. These spatio-temporal dynamics as well as those of the spindle matrix proteins Chromator and Megator together with Lamin B are summarized in S2 Fig By comparing cell cycle arrest in colchicine- and mAb 6H11-injected syncytial embryos we show that Cyclin B, Ran, and Polo, but not Greatwall, are likely constituents of the spindle matrix and that their nuclear functions may depend on spindle matrix formation.

In colchicine-injected embryos, tubulin is depolymerized but spindle matrix formation is unaffected [5]. We show that under these conditions Cyclin B, Ran, and Polo remain at enriched levels in the spindle region after NEB indicating interactions with and localization to the spindle matrix. Moreover, their spatio-temporal dynamics are maintained; for example, both Cyclin B and Polo localize transiently to the nuclear rim prior to NEB and are recruited to the kinetochores as is observed in untreated embryos. That Ran remained confined to the spindle region in association with the spindle matrix provides a plausible mechanism for how Ran-GTP/GDP gradients can be maintained at M-phase after NEB when there are no diffusion barriers. Thus, the localization and dynamics of these cell cycle factors are not solely dependent on the microtubule-based spindle apparatus.

In order to study the location and dynamics of cell cycle factors in the absence of spindle matrix formation we identified a function blocking antibody. We provide evidence that when mAb 6H11 was bound to Chromator, it prevented Chromator relocalization away from the chromosomes during prophase. Under these conditions the spindle matrix does not form as also indicated by lack of concomitant Megator relocalization from the NE suggesting a general block of spindle matrix formation. Taken together these findings suggest that the relocalization of Chromator at prophase is a key and necessary step in spindle matrix formation. It should be pointed out that other scenarios could be envisioned. However, considering the previously demonstrated high specificity of the 6H11 mAb for Chromator [7,23,36] and that the presence of the mAb 6H11 antibody does not interfere with cell cycle protein function at any point after NEB, with any checkpoint proteins, with microtubule spindle function, with cytokinesis, or with formation of the daughter nuclei these findings are all compatible with the above hypothesis.

The identification of mAb 6H11's function blocking properties is fortuitous, since at present antibody perturbation is the only avenue to prevent spindle matrix formation at mitosis and for probing its downstream consequences. It overcomes issues of functional redundancy of the known spindle matrix proteins as well as the fact that loss-of-function mutations of the key matrix proteins Chromator and Megator are early embryonic lethals (reviewed in [37]). Previously, time-lapse analysis of mitosis in S2 cells depleted of Chromator by RNAi treatment revealed several resulting phenotypes [15]. They included incomplete alignment of chromosomes at the metaphase plate, possibly due to a defective spindle assembly checkpoint, as well as of frayed and unstable microtubule spindles during anaphase [15]. The latter defect may arise due to the absence of a stabilizing interaction of Chromator with microtubules after RNAi knockdown as suggested by Yao et al. [23]. It is important to note that in the present experiments Chromator protein is at wild-type levels and that mAb 6H11 binding was not observed to cause any of the pleiotropic effects of RNAi knockdown. Thus, the 6H11 antibody's effect appears specific to Chromator relocalization from the chromosomes at prophase.

Interestingly, the spatio-temporal dynamics of the cell cycle factors (Cyclin B, Ran, and Polo) in mAb 6H11 injected embryos were very different as compared to colchicine injected embryos (summarized in S3 Fig). For example, we show that although Cyclin B levels appeared to be continuously increasing within the nucleus there was no sign of its normal enrichment at the nuclear rim, kinetochores of the condensed chromosomes, or centrosomes. Moreover, Polo was not imported into the nucleus at prophase as in wild-type and colchicine-treated embryos and was absent from the nuclear rim and the kinetochores. We speculate that the impaired spatio-temporal dynamics of spindle matrix dependent cell cycle factors is the cause of the observed cell cycle arrest as a similar phenotype can be obtained by the triple RNAi knockdown of Cyclins A, B, and B3 [13]. Thus, taken together with our previous findings for spindle matrix dependence of the spindle checkpoint proteins Mad2 and Mps1 [10,11,15] the results of the present study support the hypothesis that spindle matrix formation is a general requirement for proper dynamics and function of key cell cycle regulators during the cell cycle as well as for orchestrating the temporal loading of these factors to various nuclear locations such as the nuclear rim and kinetochores when needed. How many cell cycle factors may be involved is not known; however, as exemplified by Greatwall not all cycle factors interact with the spindle matrix and indeed may be excluded from it.

An unresolved issue in organisms undergoing open mitosis is the potential contribution of membranes to spindle form and function [38]. At the prometaphase transition in these systems the NE completely disassembles and the NE is recycled into the endoplasmic reticulum which then breaks down into vesicular or tubular membrane elements encasing the spindle forming a "spindle envelope" [39–43]. From these studies it is not clear whether the membrane association with the spindle plays a functional role or is simply a way to apportion membrane components to daughter nuclei. However, recent studies have suggested that this membranous network may contribute to spindle function by providing confinement of mitotic factors and/or working as an elastic module [38,44–47]. It has also been proposed that "spindle envelopes" might help keep the spindle region clear of large organelles that could interfere with spindle assembly [38,45]. However, based on the findings of the present study we propose an alternative hypothesis where the spindle matrix functions to exclude membranes, including "the spindle envelope", and other organelles and prevents them from entering the spindle region as well as to assist in positioning them for even distribution around the forming daughter nuclei. In support of this idea we demonstrate that Rthl1-GFP and Pdi-GFP labeled membranes coalesced around the spindle matrix as defined by Megator-mCherry without entering the spindle matrix with most membranes concentrated in the gap between the centrosomes and the spindle matrix. Importantly, this accumulation of membranes does not represent a barrier to microtubules emanating from the centrosomes and together with the finding of no diffusion barriers for molecules up to 2 MDa after NEB argues against a major role in confinement for the "spindle envelope". Nonetheless, it is likely that the interplay between microtubules, the spindle matrix, and membrane dynamics is finely tuned and mutually dependent [5,48].

At present the physio-chemical properties of the spindle matrix are poorly understood [23]; however, there are multiple examples of other membrane-less macromolecular assemblies, such as P granules, ribonucleoprotein granules/bodies (RNP droplets), nucleoli, Cajal bodies, and the centrosome, that are highly dynamic, yet cohesive (reviewed in [49,50]). Weak, repetitive interactions between the macromolecules making up these assemblies facilitate the formation of a coherent structure in the absence of a membrane, while still enabling a fluid-like micro-environment similar to that of membrane-bound organelles [49,51]. Studies have indicated that these structures can function as liquid phase micro-reactors, concentrating various protein components and accelerating the kinetics of protein-protein reactions (reviewed in [49). Recently, Jiang et al. [52] showed that the evolutionarily conserved low-complexity protein BuGZ promotes assembly of the spindle matrix as well as of the microtubule spindle apparatus in *Xenopus* egg extracts and in human cells by undergoing phase transitions or coacervation. Importantly, the study of Jiang et al. [52] strongly suggests that a spindle matrix is a general feature of cell division in vertebrates including humans. The known features of the *Drosophila* spindle matrix proteins [4,52] are consistent with the above described scenarios and they would provide a framework for future studies of how macromolecular interactions within the matrix contribute to its cohesion, functional properties, and role in the spatio-temporal regulation of cell cycle factors.

## Supporting information

S1 Fig Nuclear Cyclin B-GFP levels were unchanged in colchicine-injected embryos after NEB. (A) Image panels from a time-lapse sequence of Cyclin B-GFP (in green) and 70 kDa Dextran-TRITC (in red) after colchicine injection. Time is indicated in minutes and seconds. (B) Plots of average pixel density within the nucleus as a function of time for Cyclin B-GFP (in green) and 70 kDa Dextran-TRITC (in red). The approximate time of NEB is indicated by an arrow.
(TIF)

S2 Fig Diagrams comparing the dynamics of the spindle matrix proteins Chromator and Megator, the nuclear envelope protein Lamin B, and the cell cycle factors Cyclin B, Polo, Ran, and Greatwall during mitosis in unperturbed embryos. The diagrams are based on the results of the present study as well as of Yao et al. [5]. Chromosomes, the nuclear envelope, centrosomes, and the midbody are outlined in grey. Color intensity is proportional to relative protein levels.
(TIF)

S3 Fig Diagrams comparing the dynamics of the spindle matrix proteins Chromator and Megator, the nuclear envelope protein Lamin B, and the cell cycle factors Cyclin B, Polo, and Ran in colchicine- and mAb 6H11-arrested embryos. The diagrams are based on the results of the present study as well as of Yao et al. [5]. Chromosomes, the nuclear envelope, centrosomes, and the midbody are outlined in grey. Color intensity is proportional to relative protein levels. The asterisks indicate transient localization to the nuclear rim.
(TIF)

S1 Movie Confocal timelapse imaging of transgenically expressed Chromator-GFP (in green) and H2Av-RFP (in red) in a syncytial *Drosophila* embryo injected with Chromator mAb 6H11. Antibody was injected at interphase prior to nuclear envelope breakdown and the image sequence starts at the beginning of anaphase of the subsequent mitosis. The timelapse covers a period of 23 min 12 s. (Quicktime movie; 6.1 MB).
(MOV)

S2 Movie Confocal timelapse imaging of transgenically expressed Chromator-GFP (in green) and Tubulin-mCherry (in red) in a syncytial *Drosophila* embryo injected with Chromator mAb 6H11. Antibody was injected at interphase prior to nuclear envelope breakdown and the image sequence starts at the beginning of metaphase of the subsequent mitosis. The timelapse covers a period of 28 min 54 s. (Quicktime movie; 7.4 MB).
(MOV)

S3 Movie Confocal timelapse imaging of transgenically expressed Megator-YFP (in green) and Tubulin-mCherry (in red) in a syncytial *Drosophila* embryo injected with α-GST mAb 8C7. The antibody was injected at interphase and the image sequence starts at metaphase of the first cell cycle after the injection. The embryo completed two complete mitotic cycles and initiated a third without any observable defects as compared to wild-type embryos. The timelapse covers a period of 33 min 49 s. (Quicktime movie; 8.5 MB).
(MOV)

S4 Movie Confocal timelapse imaging of transgenically expressed Megator-YFP (in green) and Tubulin-mCherry (in red) in a syncytial *Drosophila* embryo injected with Chromator mAb 6H11. Antibody was injected at interphase prior to nuclear envelope breakdown and the image sequence starts at metaphase of the subsequent mitosis. The timelapse covers a period of 35 min 40 s. (Quicktime movie; 4.7 MB).
(MOV)

S5 Movie Confocal timelapse imaging of transgenically expressed Cyclin-B-GFP (in green) and H2Av-RFP (in red) in a syncytial *Drosophila* embryo. The timelapse covers a period of 13 min 30 s. (Quicktime movie; 7.9 MB).
(MOV)

S6 Movie Confocal timelapse imaging of transgenically expressed Cyclin-B-GFP (in green) and H2Av-RFP (in red) in a syncytial *Drosophila* embryo after colchicine injection. The timelapse covers a period of 4 min 55 s. (Quicktime movie; 7.3 MB).
(MOV)

S7 Movie Confocal timelapse imaging of transgenically expressed Cyclin B-GFP (in green) together with 70 kDa Dextran-TRITC (in red) in a syncytial *Drosophila* embryo. The timelapse covers a period of 6 min 40 s. (Quicktime movie; 6.8 MB).
(MOV)

S8 Movie Confocal timelapse imaging of transgenically expressed Cyclin-B-GFP (in green) and H2Av-RFP (in red) in a syncytial *Drosophila* embryo injected with Chromator mAb 6H11. Antibody was injected at interphase prior to nuclear envelope breakdown of the first mitosis and the image sequence starts just prior to the beginning of chromosome condensation of the subsequent mitosis. The timelapse covers a period of 15 min 48 s. (Quicktime movie; 9.5 MB).
(MOV)

S9 Movie Confocal timelapse imaging of transgenically expressed Ran-Venus (in green) and Tubulin-mCherry (in red) in a syncytial *Drosophila* embryo. The timelapse covers a period of 9 min 0 s. (Quicktime movie; 9.5 MB).
(MOV)

S10 Movie Confocal timelapse imaging of transgenically expressed Ran-Venus (in green) and Tubulin-mCherry (in red) in a syncytial *Drosophila* embryo after colchicine injection. The timelapse covers a period of 9 min 48 s. (Quicktime movie; 6.1 MB).
(MOV)

S11 Movie Confocal timelapse imaging of transgenically expressed Ran-Venus (in green) and Tubulin-mCherry (in red) in a syncytial *Drosophila* embryo injected with Chromator mAb 6H11. Antibody was injected at interphase prior to nuclear envelope breakdown of the first mitosis which proceeds normally; however the second round of mitosis is arrested. The timelapse covers a period of 41 min 0 s. (Quicktime movie; 8.3 MB).
(MOV)

S12 Movie Confocal timelapse imaging of transgenically expressed Polo-GFP in a syncytial *Drosophila* embryo. The timelapse covers a period of 12 min 53 s. (Quicktime movie; 6.7 MB).
(MOV)

S13 Movie Confocal timelapse imaging of transgenically expressed Polo-GFP in a syncytial *Drosophila* embryo after colchicine injection. The timelapse covers a period of 6 min 42 s. (Quicktime movie; 5.4 MB).
(MOV)

S14 Movie Confocal timelapse imaging of transgenically expressed Polo-GFP in a syncytial *Drosophila* embryo injected with Chromator mAb 6H11. Antibody was injected at interphase prior to nuclear envelope breakdown of the first mitosis and the image sequence starts just prior to the beginning of chromosome condensation of the subsequent mitosis. The timelapse covers a period of 15 min 0 s. (Quicktime movie; 5.1 MB).
(MOV)

S15 Movie Confocal timelapse imaging of transgenically expressed Greatwall-GFP (in green) together with 70 kDa Dextran-TRITC (in red) in a syncytial *Drosophila* embryo. The timelapse covers a period of 21 min 20 s. (Quicktime movie; 9.3 MB).
(MOV)

S16 Movie Confocal timelapse imaging of transgenically expressed Rtnl-GFP (in green) and Megator-mCherry (in red) in a syncytial *Drosophila* embryo. The timelapse covers a period of 7 min 6 s. (Quicktime movie; 8.7 MB).
(MOV)

S17 Movie Confocal timelapse imaging of transgenically expressed Pdi-GFP (in green) and Tubulin-mCherry (in red) in a syncytial *Drosophila* embryo. The timelapse covers a period of 11 min 35 s. (Quicktime movie; 9.5 MB).
(MOV)

S18 Movie Confocal timelapse imaging of transgenically expressed Pdi-GFP (in green) together with 70 kDa Dextran-TRITC (in red) in a syncytial *Drosophila* embryo after colchicine injection. The timelapse covers a period of 3 min 9 s. (Quicktime movie; 4.8 MB).
(MOV)
