# Supplementary figures and images for: Evidence for a role of spindle matrix formation in cell cycle progression by antibody perturbation
Source: PLoS One. 2018 Nov 28;13(11):e0208022. doi: 10.1371/journal.pone.0208022 (PMC6261614; doi:10.1371/journal.pone.0208022)

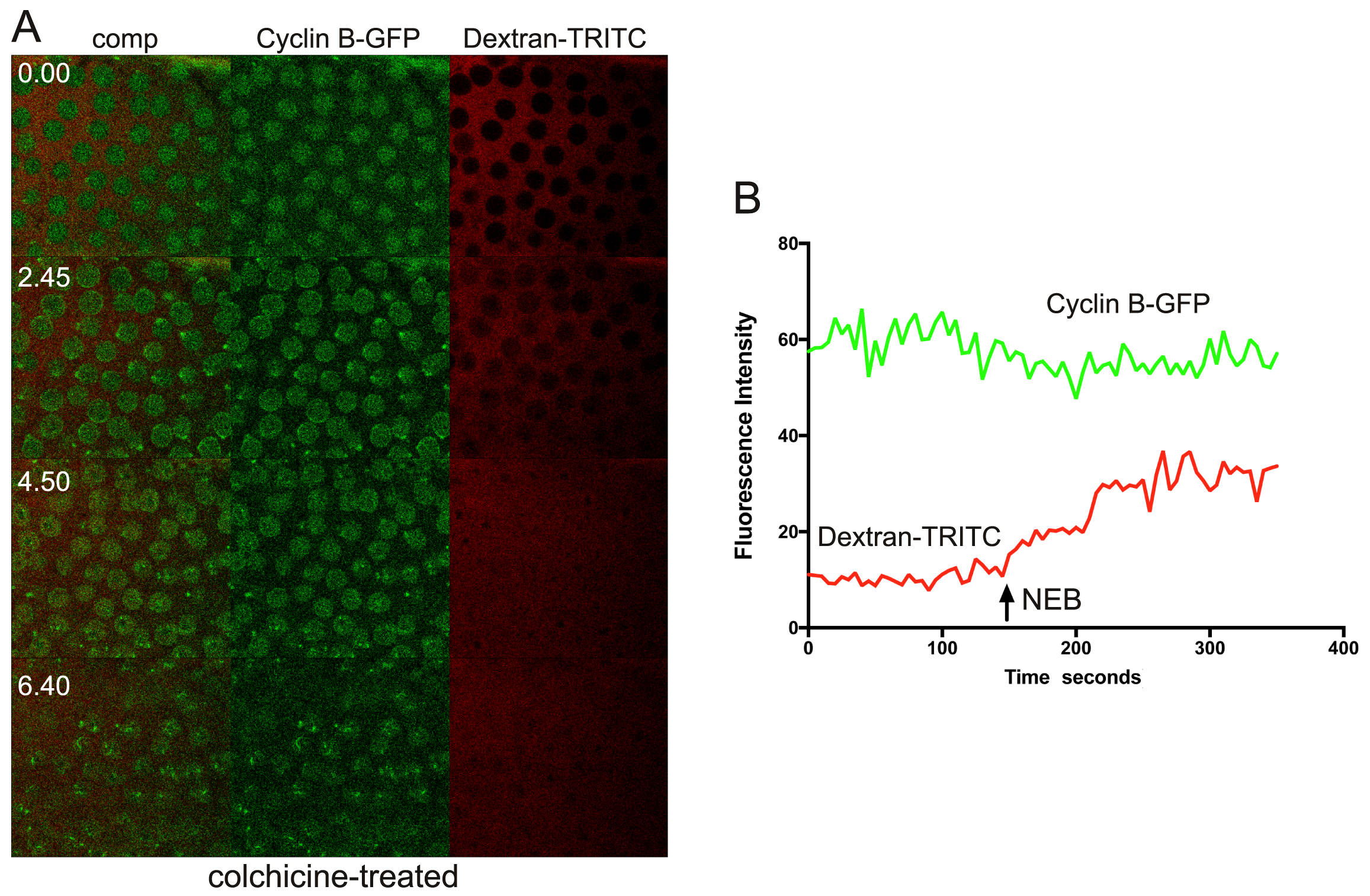

Supplement: S1 Fig — (A) Image panels from a time-lapse sequence of Cyclin B-GFP (in green) and 70 kDa Dextran-TRITC (in red) after colchicine injection. Time is indicated in minutes and seconds. (B) Plots of average pixel density within the nucleus as a function of time for Cyclin B-GFP (in green) and 70 kDa Dextran-TRITC (in red). The approximate time of NEB is indicated by an arrow. (TIF) [file pone.0208022.s001.tif]

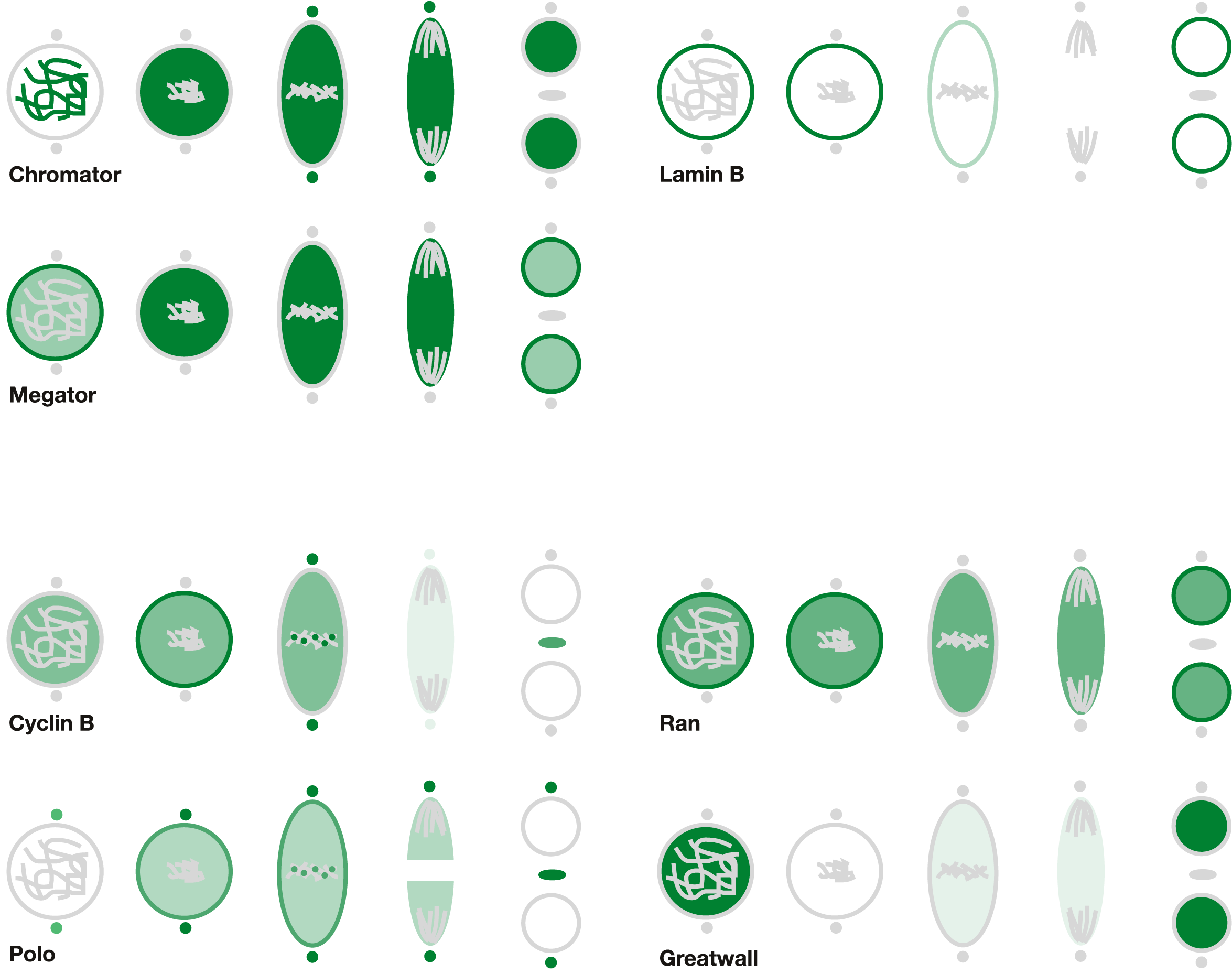

Supplement: S2 Fig — The diagrams are based on the results of the present study as well as of Yao et al. [5]. Chromosomes, the nuclear envelope, centrosomes, and the midbody are outlined in grey. Color intensity is proportional to relative protein levels. (TIF) [file pone.0208022.s002.tif]

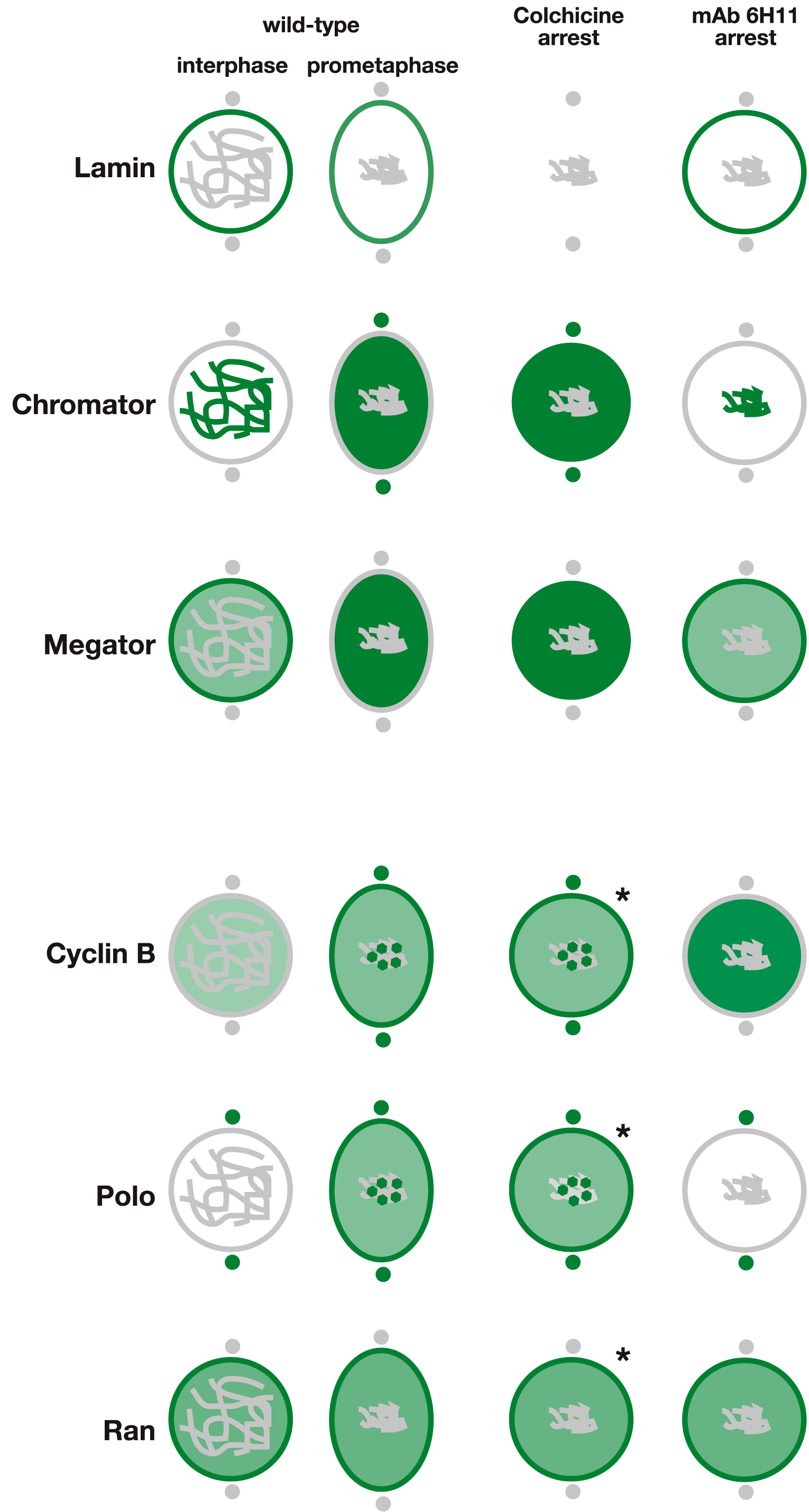

Supplement: S3 Fig — The diagrams are based on the results of the present study as well as of Yao et al. [5]. Chromosomes, the nuclear envelope, centrosomes, and the midbody are outlined in grey. Color intensity is proportional to relative protein levels. The asterisks indicate transient localization to the nuclear rim. (TIF) [file pone.0208022.s003.tif]
